# Comparison of ADVIA Centaur ultra-sensitive and high-sensitive assays for troponin I in serum

**DOI:** 10.1016/j.plabm.2022.e00293

**Published:** 2022-07-12

**Authors:** Joško Osredkar, Teja Fabjan, Kristina Kumer, Jure Tršan, Laura Poljančič, Miha Košir, Pia Vovk, Nada Snoj, Petra Finderle, Hugon Možina

**Affiliations:** a University Medical Centre Ljubljana, Institute of Clinical Chemistry and Biochemistry, Zaloška c.002, 1000, Ljubljana, Slovenia; b University of Ljubljana, Faculty of Pharmacy, Aškerčeva 7, 1000, Ljubljana, Slovenia; c University of Ljubljana, Medical Faculty, Vrazov trg 2, 1000, Ljubljana, Slovenia; d University Medical Centre Ljubljana, Institute of Radiology, Zaloška c.002, 1000, Ljubljana, Slovenia; e University Medical Centre Ljubljana, Division of Internal Medicine, Medical Emergency Unit, Zaloška c.002, 1000, Ljubljana, Slovenia; f University Medical Centre Ljubljana, Division of Surgery, Department of Anaesthetics and Surgical Intensive Care, Zaloška c.002, 1000, Ljubljana, Slovenia

**Keywords:** Acute coronary syndrome, Myocardial infarction, Troponin I, Verification, acute coronary syndrome, ACS, acute myocardial infarction, AMI, confidence interval, CI, cardiac troponin I, cTnI, chronic obstructive pulmonary disease, COPD, coefficient of variation, CV, limit of detection, LoD, limit of blank, LoB, functional sensitivity, LoQ, high-sensitive cTnI, hs-cTnI, upper reference of normal, URL, ultra-sensitive cTnI, us-cTnI

## Abstract

Cardiac troponin I (cTnI) is a standard biomarker for the diagnosis of acute myocardial infarction (AMI). While older, ultra-sensitive cTnI (us-cTnI) assays use the 99th percentile as the reference threshold, newer high-sensitive cTnI (hs-cTnI) assays use the limit of detection or functional sensitivity instead. However, little has been done to systematically compare these two methods. The present study also served as a validation of hs-cTnI in our laboratory. Here, we compared the results obtained from the blood serum obtained from 8810 patients using the us-cTnI and the hs-cTnI assays run in tandem on the ADVIA Centaur XP analyser. We found that in 2279 samples the concentration of cTnI measured with the ultra-sensitive method was below the detection limit, while with the high-sensitive method, only 540 were below the detection limit. We also compared results from these assays with the ultimate diagnosis of a subset of individuals. The analysis of the results below cut-off with the ultra-sensitive method showed that this method would not detect 96 cases related to heart disorder. Overall, the main finding of our research is that hs-cTnI is the preferable option and is able to be deployed effectively in the laboratory setting.

## Highlights

• Measuring levels of cTnI is an important method of identifying heart-related disorders in the hospital setting.
• In the present study, the ultra-sensitive method did not detect 96 cases related to heart disorder.
• The high-sensitive cTnI assay the preferable option and is able to be deployed effectively in the laboratory setting.

## 1 Introduction

In western countries, the mortality for coronary heart disease has gradually declined over the last few decades. But this condition still causes about one-third of all deaths in people older than 35 years. The incidence and mortality for acute coronary syndrome (ACS) remain high [1]. Additionally, suspected acute myocardial infarction (AMI) is a burden for emergency departments worldwide [2]. Rapid ACS and AMI diagnostic tools are necessary in order to give appropriate and timely care to patients [3].

Diagnosis of AMI is based on clinical assessment, electrocardiography, and the presence of the cardiac regulatory protein troponin I (cTnI) [4]. Over the years, clinical practice has gradually changed with improving sensitivity of cTnI assays [5]. The new standard for the high-sensitive cardiac troponin assay (hs-cTnI) is defined as the ability to detect cTnI precisely with a coefficient of variation <10% at or below the 99th percentile upper reference of normal (URL) and is thus detectable in >50% of normal healthy individuals [6]. Older troponin I diagnostic algorithms used the 99th percentile as the reference threshold, but the new hs-cTnI takes the limit of detection (LoD) or functional sensitivity (or limit of quantification; LoQ) instead, and this improved analytical performance is giving us the possibility to classify methods based on the ratio between the 99th percentile and LoD [7]. But when using any available method it is important to be aware of assay problems, pre-analytical, and analytical factors that may result in false-positive troponin measurements [8].

The purpose of the present study was to introduce a the new hs-cTnI method into routine laboratory work and validate it by comparing it to the older, ultra-sensitive cTnI (us-cTnI) method. Further, we aimed to interpret the results from patients processed at the emergency department where the ultra-sensitive method gave different results, at time, from the high-sensitive method. The final diagnosis was compared against the result of both methods in order to understand which method if more likely to yield the correct result.

## 2 Methods

### 2.1 Patient enrolment

A total of 10,563 blood samples, with an order for cTnI, were collected at the University Medical Centre Ljubljana (UMCL) in Ljubljana, Slovenia, from April 1, 2019 until June 4, 2019.1753 samples are not included in these analyses because only one assay was used. Ultimately, in 8810 of the collected samples, blood serum was analysed with the standard us-cTnI assay in parallel with the new hs-cTnI method. From this large number of compared results, we selected 360 results in the concentration ranges described by the recommendations of the Clinical & Laboratory Standards Institute (CLSI) standard [9] for analysis. Basic demographic information (age and gender) was collected, along with the date and hour of collection (data not shown). This study was approved by the UMCL Ethics Committee. Informed consent was not required because blood collection is a routine part of hospital intake at UMCL.

### 2.2 Sample collection

Blood samples were obtained by routine venepuncture in accordance with the standard procedure at UMCL. The total blood volume was 4 ml in a plain tube without an anticoagulant. All samples were processed according to hospital protocols. Specifically, samples were centrifuged at 1500×*g* for 10 min to obtain serum.

### 2.3 cTnI assays

The us-cTnI and hs-cTnI assays were performed simultaneously on the ADVIA Centaur XP analyser (Siemens Healthcare). The new ADVIA Centaur hs-cTnI method is a three-site sandwich immunoassay using direct chemiluminometric technology and the fully automated Centaur XP platform. All samples were run at the same time and in accordance with the manufacturer's recommendations. All assay runs were within laboratory specified parameters, including quality control limits. Confirmed results were sent to the prescribing doctor. The comparison of quality control parameters for both methods revealed differences in the limit of detection (LoD) and limit of quantification (LoQ). For us-cTnI method LoD was 0.006 μg/L and the LoQ was 0.017 μg/L while for hs-cTnI the LoD was 0.0022 μg/L and the LoQ was 0.0025 μg/L, as provided by the manufacturer.

### 2.4 Data analysis

Data analysis was performed with Medcalc, version 19.1. A linear regression analysis was performed for the assay comparison. Descriptive statistics are used elsewhere. Statistical significance was defined as *P* < 0.05.

## 3 Results

### 3.1 Introduction of a new, high-sensitive cTnI method into routine laboratory work

The precision profile of the hs-cTnI method was estimated by measuring control material (Liquichek cardiac markers plus control) at three different levels to display low (approximately 37 μg/L), intermediate (approximately 4.860 μg/L) and high (approximately 12.600 μg/L) cTnI concentrations. The within-run and between-run precision was measured using a 6x6 protocol. The final data was reported in terms of coefficient of variation (CV%).

Results of the precision studies using QC material with low, medium and high cTnI values are summarized in Table 1. The within-run coefficient of variation was between 1.1% and 1.9% and the between-run coefficient of variation was between 1.3% and 2.7%. The total coefficient of variation ranged between 1.3% and 2.7%.

**Table 1:** Quality control data for hs-cTnI method.

		Repeatability (within-run)		Intermediate precision (between –run)		Reproducibility (total precision)
μg/l	6 x 6	QC data (provider)	Our results	QC data (provider)	Our results	Our results
**Level 1**	37	2.8	1.9	3.7	2.7	2.7
**Level 2**	4.900	1.6	1.1	2.6	1.3	1.3
**Level 3**	12.700	1.5	1.3	2.7	1.5	1.5

QC data are presented as CV % (Coefficient of variation).

Calculations were performed using the EP15-A3 protocol [10] and are available as a Supplementary file.

Analytical performance of a laboratory method is defined with the LoD, limit of blank (LoB), LoQ, and the 99th percentile of a healthy reference population [[11], [12], [13]], as described in. These two methods have different analytical performance and have all the calculated ratio between 99th percentile and LoD greater than 1 (us-cTnI = 6.67; hs-cTnI method = 21.81).

**Fig. 1:**
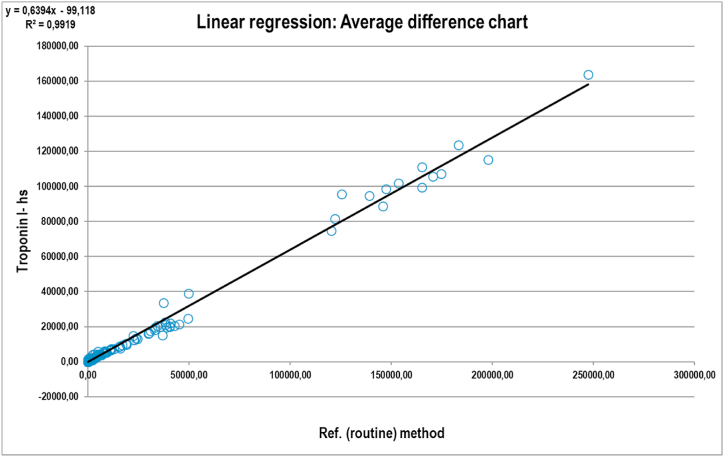
Linear regression line of 360 compared determinations.

**Fig. 2:**
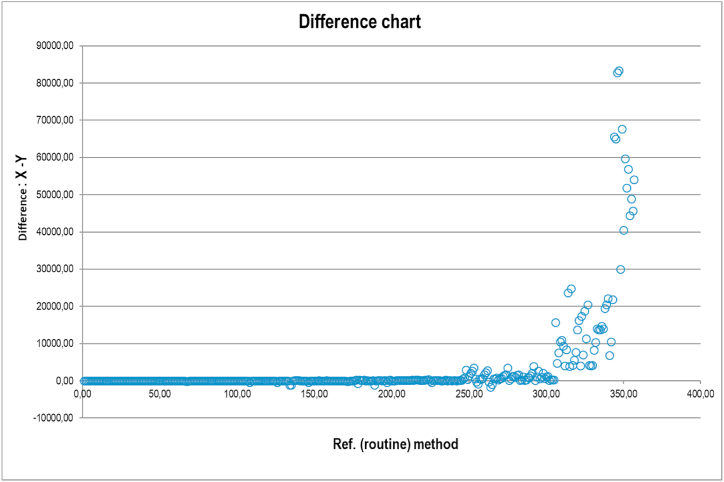
Calculated differences between us-cTnI and hs-cTnI.

The calculation showed the following regression equation: Y = 0.639X - 99.118 with value r = 0.996 and all coefficients are within allowed confidence interval (CI(lower) = −487.71; CI(upper) = 363.59). The calculated bias corresponds to the biological variability of us-cTnI also [14]. The linear regression is shown in Fig. 1, while the differences between the two methods are shown in Fig. 2.

**Fig. 3:**
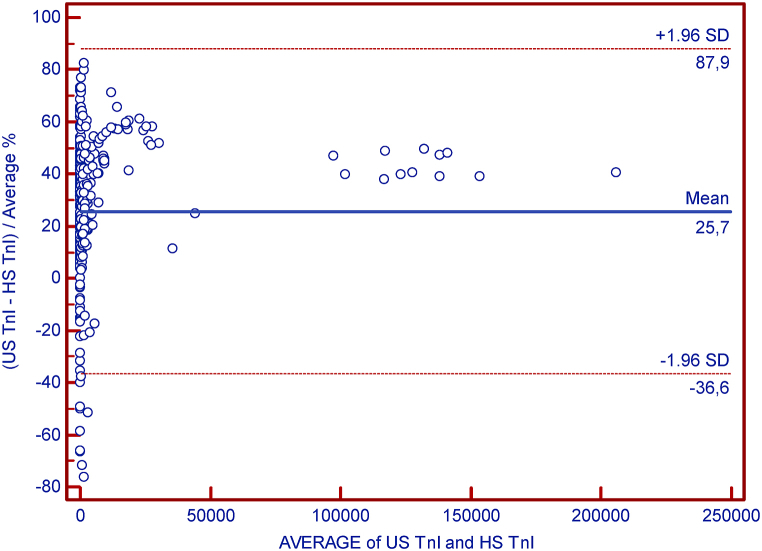
Bland-Altman plot of cTnI values.

**Fig. 4:**
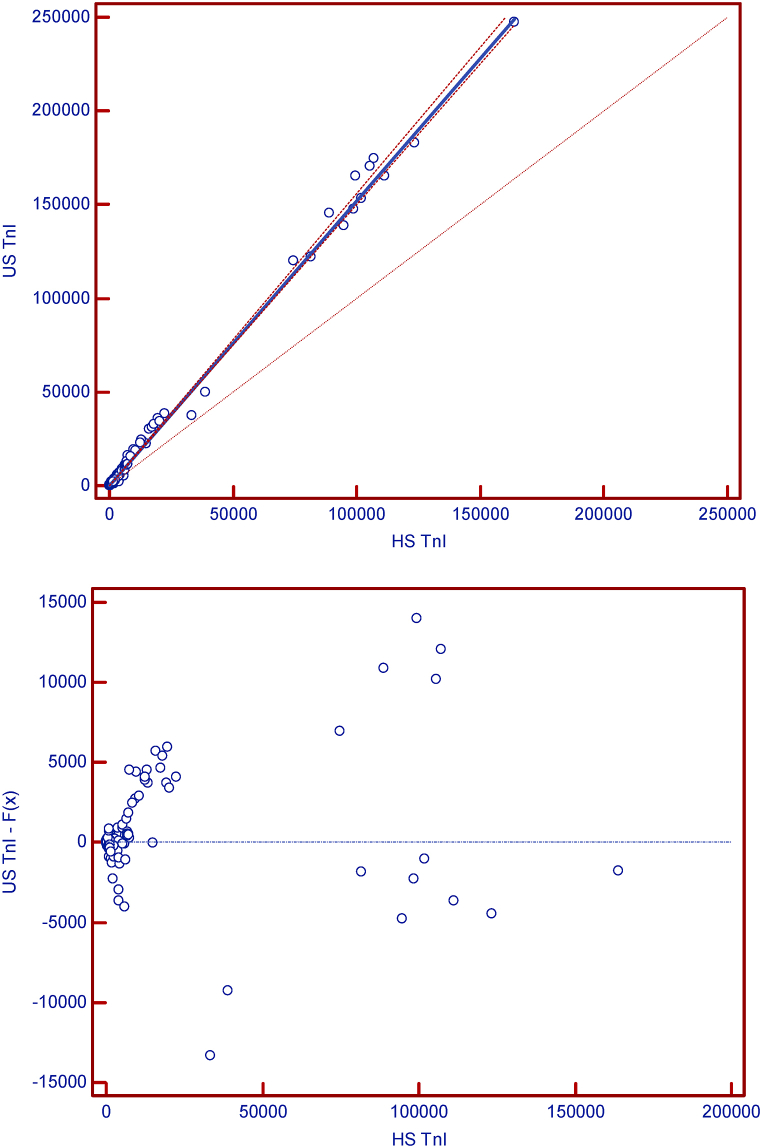
Passing-Bablok regression analyses of two methods for cTnI. (A)Scatter diagram with regression line and confidence bands for regression line. (B) Residual plot presents distribution of difference around fitted regression line.

For a more detailed analysis, 360 serum samples were taken from subjects treated in the emergency department. We excluded outliers in the calculation and thus performed the calculation on the final 324 samples. Concordance was assessed with a Passing-Bablok regression analysis, whereas absolute bias was assessed using a Bland-Altman plot analysis. Fig. 3, Fig. 4 show the results graphically.

As can be seen in all presented figures, higher scattering occurs at higher concentrations, even outside the limits, but these values are no longer clinically relevant. Overall, we concluded that the new hs-cTnI method is comparable to the us-cTnI method.

### 3.2 The hs-cTnI method does a better job predicting diagnosis of a cardiac event than the us-cTnI method

Due to the large number of samples, in the next phase we restricted ourselves to samples from the emergency department (3068 samples) and made further calculations. We reviewed the final diagnoses that patients received and compared them against the predictions of the cTnI assays. In 164 cases, we found that the cut off values of troponin measured by the hs-CTnI method and the us-cTnI did not match, such that the us-cTnI method gave a negative result while the hs-cTnI gave a positive result. Ultimate diagnoses defining cardiovascular disease occurred in 58.5% (96/164 cases). In the remaining cases, 25% (41/164) had a definitive diagnosis of non-cardiac origin, and 16.5% (27/164) with further investigation of the final cause were not found.

Among diagnoses of cardiovascular disease, conditions associated with the vascular system occurred in 27% (26/96), while cardiac events occurred in 72.9% (70/96). Among vascular diseases, arterial hypertension (73.1%; 19/26) was most commonly observed, followed by pulmonary embolism (11.5%; 3/26), orthostatic hypotension (11.5%; 3/26), and deep vein thrombosis (3.8%; 1/26). Among the conditions where patients suffered a cardiac event, the frequency of worsening heart failure, which occurred in a half of the cases (51.4%; 36/70), was notable. Worsening of heart failure also included those conditions in which patients simultaneously suffered from exacerbation of chronic obstructive pulmonary disease (COPD), the latter being 16% (6/36). The remaining cardiac events were arrhythmias in 34.3% (24/70), unstable angina (UAP) in 8.6% (6/70) and acute myocardial infarction in 5.7% (4/70).

Among non-cardiac conditions, the most commonly observed pneumonia was 33.3% (9/27), followed by gastritis in 22.2% (6/27), COPD in 18.5% (5/27), sepsis in 14.8% (4/27), syncope 14.8% (4/27), hyperkalaemia 14.8% (4/27), pulmonary oedema in 11.1% (3/27), pericardial effusion in 7.4% (2/27), obstruction in 7.4% (2/27), dehydration in 3.7% (1/27) and cervical spine defect with paraesthesia in 3.7% (1/27).

## 4 Discussion

The initial evaluation of patients presenting with acute chest pain is challenging because of the heterogeneity of underlying conditions and the consequences of inappropriate management [15]. Patients with chest pain suggestive of myocardial infarction represent up to 10% of all presentations to emergency departments [16,17]. However, only 5%–10% of these patients will have a confirmed diagnosis of myocardial infarction on discharge [18]*.* Troponin testing is commonly used to diagnose or exclude AMI in patients presenting to the emergency department with symptoms compatible with ACS, but it may be elevated in many stable and unstable cardiac and non-cardiac conditions.

A relevant question involves expectations that we may have when switching our current central laboratory assays to the next generation of hs-cTnI assays. The use of new generation high sensitivity cTnI assays have lowered the diagnostic threshold (specificity). This is clearly shown by the results of our comparison, when we determined 2275 results below the detection limit by the us-cTnI method, whereas by the new hs-cTnI method, only 540 were below the detection limit.

When looking at the comparison between us-cTnI and hs-cTnI results, we can see that 96 cases where a cardiac diagnosis was ultimately made would have been missed if only the us-cTnI method was used. This suggest that prior to the implementation of hs-cTnI assays, there were likely cardiac events that were missed, and the patients probably mistakenly sent home from emergency department. Altogether, these data suggest that the hs-cTnI is the preferable option over ultra-sensitive assays for troponin I. Furthermore, hs-cTnI is able to be deployed effectively in the laboratory setting.

## Author contributions

All authors met the criteria for this journal's authorship credit as set forth by the author's guide. Each authors' name is given next to the appropriate category; Study conception and design: JO; Laboratory Analysis: NS, PF; Statistical Analysis: TF, KK, JT; Providing clinical data: MK, PV; Interpretation of data: JO, LP, HM; Drafting of manuscript: JO; Critical revision: HM.

## Authors declare NO conflict of interest

The introduction of the new method for troponin I (replacement of the old one) was due to the availability of a new generation of reagent for troponin I. At the University Clinical Centre, we approached the replacement of the existing analytical systems in such a way that both methods were run in parallel on the same analyser over a period of three months. All results were firstly reviewed in terms of technical requirements, then from a laboratory point of view and finally from a clinical point of view. All of this has been merged into a pre-existing manuscript.

## Declaration of competing interest

The authors have not applied for any patents relating to the content of the manuscript, and did not receive reimbursements, fees, funding, or salary from an organization that holds or has applied for a patent relating to the content of the manuscript. The authors declare that they have no conflict of financial and nonfinancial interest relating to this work.

## References

[1] Sanchis-Gomar F., Perez-Quilis C., Leischik R., Lucia A. (2016). Epidemiology of coronary heart disease and acute coronary syndrome. Ann. Transl. Med..

[2] Niska R., Bhuiya F., Xu J. (2010). National hospital ambulatory medical care survey: 2007 Emergency department summary. Natl Health Stat Report.

[3] Goodacre S., Thokala P., Carroll C., Stevens J.W., Leaviss J., Al Khalaf M. (2013). Systematic review, meta-analysis and economic modelling of diagnostic strategies for suspected acute coronary syndrome. Health Technol. Assess..

[4] Thygesen K., Alpert J.S., Jaffe A.S., Chaitman B.R., Bax J.J., Morrow D.A. (2018). Fourth universal definition of myocardial infarction (2018). Circulation.

[5] Thygesen K., Alpert J.S., Jaffe A.S., Simoons M.L., Chaitman B.R., White H.D. (2012). Third universal definition of myocardial infarction. Circulation.

[6] Apple F.S., Collinson P.O. (2012). Analytical characteristics of high-sensitivity cardiac troponin assays. Clin. Chem..

[7] Lippi G., Sanchis-Gomar F. (2018). Ultra-sensitive” cardiac troponins: requirements for effective implementation in clinical practice. Biochem. Med..

[8] Jaffe A.S. (2012). Troponin-past, present, and future. Curr. Probl. Cardiol..

[9] Budd J.R., Durham A.P., Gwise T.E., Iriarte B., Kallner A., Linnet K. (2013).

[10] Carey N.R., Durham A.P., Hauck W.W., Kallner A., Kondratovich M.V. (2014).

[11] Armbruster D.A., Pry T. (2008). Limit of blank, limit of detection and limit of quantitation. Clin. Biochem. Rev..

[12] Apple F.S., Jaffe A.S., Collinson P., Mockel M., Ordonez-Llanos J., Lindahl B. (2015). IFCC educational materials on selected analytical and clinical applications of high sensitivity cardiac troponin assays. Clin. Biochem..

[13] Apple F.S. (2009). A new season for cardiac troponin assays: it's time to keep a scorecard. Clin. Chem..

[14] Ricos C, Alvarez V, Cava F, Garcia-Lario J, Hernandez A, Jimenez C, et al. Desirable Specifications for Total Error, Imprecision, and Bias, Derived from Intra- and Inter-individual Biologic Variation. Available at: https://www.westgard.com/biodatabase1.htm.Accessed December 18th 2020.

[15] Eggers K.M., Jernberg T., Ljung L., Lindahl B. (2018). High-Sensitivity cardiac troponin-based strategies for the assessment of chest pain patients-a review of validation and clinical implementation studies. Clin. Chem..

[16] Goodacre S., Cross E., Arnold J., Angelini K., Capewell S., Nicholl J. (2005). The health care burden of acute chest pain. Heart.

[17] Mockel M., Searle J., Muller R., Slagman A., Storchmann H., Oestereich P. (2013). Chief complaints in medical emergencies: do they relate to underlying disease and outcome? the Charité Emergency Medicine Study (CHARITEM). Eur. J. Emerg. Med..

[18] Bandstein N., Ljung R., Johansson M., Holzmann M.J. (2014). Undetectable high-sensitivity cardiac troponin T level in the emergency department and risk of myocardial infarction. J. Am. Coll. Cardiol..

